# The epidemiology of chronic kidney disease (CKD) in rural East Africa: A population-based study

**DOI:** 10.1371/journal.pone.0229649

**Published:** 2020-03-04

**Authors:** Anthony N. Muiru, Edwin D. Charlebois, Laura B. Balzer, Dalsone Kwarisiima, Assurah Elly, Doug Black, Samuel Okiror, Jane Kabami, Mucunguzi Atukunda, Katherine Snyman, Maya Petersen, Moses Kamya, Diane Havlir, Michelle M. Estrella, Chi-yuan Hsu

**Affiliations:** 1 University of California, San Francisco, California, United States of America; 2 Kidney Health Research Collaborative, San Francisco Veterans Affairs Medical Center, San Francisco, California, United States of America; 3 University of Massachusetts, Amherst, Massachusetts, United States of America; 4 Infectious Diseases Research Collaboration, Kampala, Uganda; 5 Kenya Medical Research Institute, Nairobi, Kenya; 6 School of Public Health, University of California, Berkeley, California, United States of America; 7 Makerere University, Kampala, Uganda; Loyola University Chicago, UNITED STATES

## Abstract

**Background:**

Chronic kidney disease (CKD) may be common among individuals living in sub-Saharan Africa due to the confluence of CKD risk factors and genetic predisposition.

**Methods:**

We ascertained the prevalence of CKD and its risk factors among a sample of 3,686 participants of a population-based HIV trial in rural Uganda and Kenya. Prevalent CKD was defined as a serum creatinine-based estimated glomerular filtration rate <60 mL/min/1.73m^2^ or proteinuria (urine dipstick ≥1+). We used inverse-weighting to estimate the population prevalence of CKD, and multivariable log-link Poisson models to assess the associations of potential risk factors with CKD.

**Results:**

The estimated CKD prevalence was 6.8% (95% CI 5.7–8.1%) overall and varied by region, being 12.5% (10.1–15.4%) in eastern Uganda, 3.9% (2.2–6.8%) in southwestern Uganda and 3.7% (2.7–5.1%) in western Kenya. Risk factors associated with greater CKD prevalence included age ≥60 years (adjusted prevalence ratio [aPR] 3.5 [95% CI 1.9–6.5] compared with age 18–29 years), HIV infection (aPR 1.6 [1.1–2.2]), and residence in eastern Uganda (aPR 3.9 [2.6–5.9]). However, two-thirds of individuals with CKD did not have HIV, diabetes, or hypertension as risk factors. Furthermore, we noted many individuals who did not have proteinuria had dipstick positive leukocyturia or hematuria.

**Conclusion:**

The prevalence of CKD is appreciable in rural East Africa and there are considerable regional differences. Conventional risk factors appear to only explain a minority of cases, and leukocyturia and hematuria were common, highlighting the need for further research into understanding the nature of CKD in sub-Saharan Africa.

## Introduction

Chronic kidney disease (CKD) is an important global public health problem—estimated to affect 10% of the world’s population [1]. In the United States, African Americans have several fold higher rates of kidney failure than European Americans [2, 3]. Individuals living in sub-Saharan Africa may have a heightened CKD risk similar to African Americans due to shared genetic susceptibility, such as that arising from apolipoprotein L1 (*APOL1*) gene risk variants and sickle cell trait [4–7]. CKD risk may be further exacerbated by HIV infection and its treatment [8], the rising prevalence of hypertension [9], and diabetes [10], as well as the use of potentially nephrotoxic traditional herbal medicines [11–13]. In sub-Saharan Africa, life-saving renal replacement therapy (dialysis or kidney transplantation) is not readily available, and consequently, those who progress to end-stage renal disease (ESRD) die prematurely. For the few who obtain access to renal replacement therapy, the cost often represents an enormous financial burden on individuals and their families [14, 15]. Therefore, CKD has far-reaching implications for the health and welfare of people living in this region.

However, little is known regarding CKD and its epidemiology in sub-Saharan Africa. The existing literature is limited by small sample sizes and lack of representation of participants from rural areas where the vast majority of the population resides [16]. A recent systematic review of CKD epidemiology in sub-Saharan Africa concluded that the currently published studies are mostly of low quality [17]. To improve our understanding of CKD in sub-Saharan Africa, we investigated the prevalence of CKD and associated risk factors in a representative community-based sample of rural East Africans.

## Methods

### Design and study population

**Fig 1:**
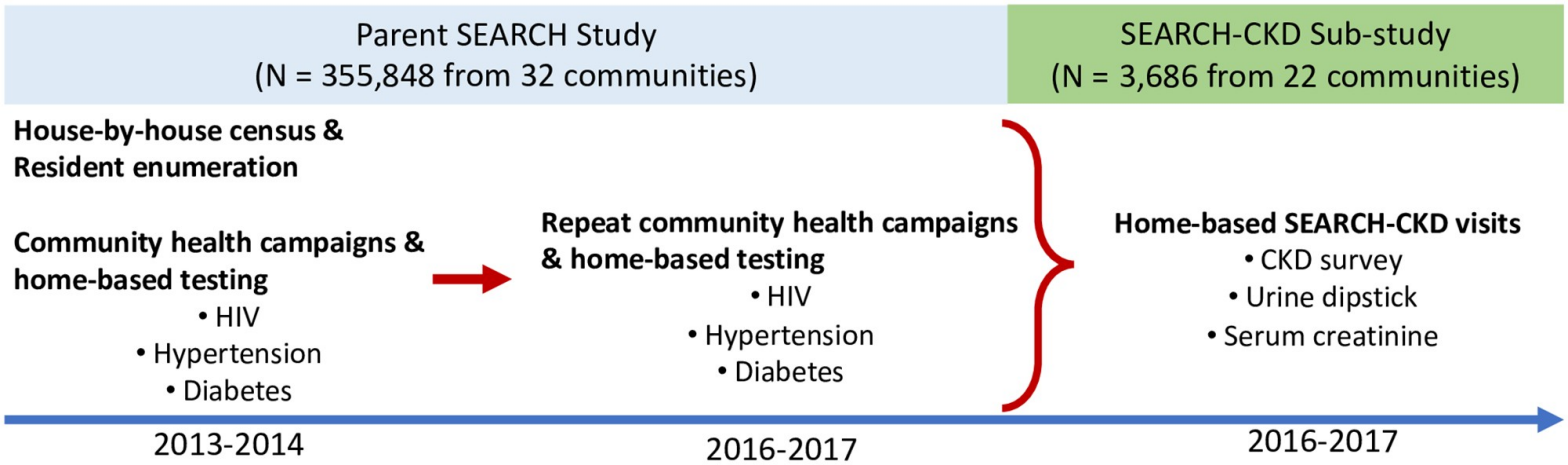
Timeline and study activities for the sustainable East Africa research in Community Health (SEARCH) study sites, and SEARCH-CKD sub-study.

This is a population-based cross-sectional study leveraging the infrastructure of the Sustainable East Africa Research in Community Health (SEARCH, NCT01864603) trial [18]. SEARCH was a 32-community cluster-randomized controlled trial with 355,848 participants living in 10 communities in eastern Uganda, 10 in southwestern Uganda and 12 in western Kenya [18]. In each of the 32 rural communities, a house-to-house census enumeration of household members was conducted from 2013 to 2014, followed by mobile 2-week multi-disease community health campaigns, which was then followed by home-based testing for non-participants (Fig 1). Participants underwent screening for HIV, hypertension and diabetes. HIV-positive persons and those screening positive for hypertension or diabetes were then referred for diagnosis and treatment. Repeat population-based testing was conducted annually in intervention communities and again in all communities among individuals who had not moved away at study close in 2016–2017 (87%). SEARCH found that a multi-disease, community-based approach to universal HIV test-and-treat approach reduced HIV incidence and improved community health at a population level [18].

To estimate the prevalence of CKD and understand its risk factors in rural East Africa, we conducted a home-based sub-study (named SEARCH-CKD) during parent study year 3 (2016–2017). We utilized a sampling framework (established by the parent study in 2013) where 100 households with at least one HIV-positive adult and 100 households without any HIV-positive adults were randomly selected to participate in each study community [19]. For the SEARCH-CKD sub-study, one HIV-positive adult was randomly selected from HIV-positive households, and one HIV-negative adult was randomly selected from HIV-negative households. During SEARCH-CKD specific home visits, participants underwent a health survey with emphasis on potential CKD risk factors as well as blood and urine sample collection at their household. The SEARCH CKD sub-study supported the survey and assessments of kidney health including serum creatinine and dipstick urinalysis which were not part of the parent SEARCH study (Fig 1).

### Kidney function assessments

Collected blood samples were transported to a local processing center. After processing, serum samples were then transferred to a central laboratory in each of the three study regions (eastern Uganda, southwestern Uganda and western Kenya) where serum creatinine was measured by the Jaffe method, traceable to isotope dilution mass spectrometry (IDMS) reference on the Cobas Integra 400 Plus (Roche Diagnostics, South San Francisco, CA, USA) and DRI-CHEM NX500i (Fujifilm, Tokyo, Japan) platforms in Uganda and Kenya, respectively. The resultant standardized serum creatinine values were used to estimate glomerular filtration rate (eGFR) using the CKD-EPI equation [20], without the black race coefficient given concerns that this may misclassify Africans [21–23].

We also conducted sensitivity analysis using the CKD-EPI equation with the black race coefficient [20].

### Determination of proteinuria

Urine dipstick analysis was performed using Uriscan Gen 10 Healgen URS-10T strips (Healgen Scientific, LLC, Bellaire, TX, USA) in Uganda, and Uriscan SGL strips (Biosys Laboratories, Inc., South Pasadena, CA, USA) were used in Kenya. Dipstick proteinuria results were interpreted by trained study personnel as “negative,” “trace,” “1+”, “2+” or “≥3+”.

### Additional dipstick urinalysis abnormalities

We also gathered concurrent data on dipstick urinalysis hematuria and leukocyte esterase positivity (urine dipstick trace or greater). To reduce the chances of urinalysis abnormalities simply reflecting urinary tract infections, we did not count cases of leukocyte esterase positivity in which nitrite was also positive during the analysis (only 5% of dipstick urinalysis positive for leukocyte esterase were also positive for nitrite).

### CKD prevalence

We defined CKD as an eGFR <60 mL/min/1.73m^2^ or presence of proteinuria (1+ or greater dipstick proteinuria). Individuals with CKD were further classified into stages 1–5 according to the 2012 Kidney Disease: Improving Global Outcomes [24] CKD guidelines: Stage 1: eGFR ≥90mL/min/1.73m^2^ and proteinuria; stage 2: eGFR 60–89 mL/min/1.73m^2^ and proteinuria; stage 3–5: eGFR <60 ml/min/1.73m^2^ regardless of proteinuria [25].

### Exposures

Exposures of interest including hypertension, diabetes mellitus, and HIV infection were ascertained at the parent SEARCH baseline (2013–2014) and follow-up (2016–2017) visits (Fig 1) [18]. Blood pressures were measured using automated oscillometric devices. Hypertension was defined as a systolic blood pressure ≥140 mm Hg or diastolic blood pressure ≥90 mm Hg or self-reported current use of anti-hypertensive medications [26]. Participants who met criteria for hypertension during initial blood pressure measurement underwent two repeat measurements one minute apart to confirm the diagnosis of hypertension. Glucose was measured using a finger-prick point-of-care non-fasting random blood glucose test (Optium-Abbott, Alameda, CA, USA). Diabetes mellitus was defined as a random blood sugar >200 mg/dL (11.1 mmol/L) [26]. HIV serostatus was determined by serial testing algorithms using point-of-care testing devices as previously described [18]. Approximately 2% of our study participants were missing measurements for hypertension, diabetes mellitus, or HIV.

Additional exposures including self-reported use of nonsteroidal anti-inflammatory drugs (NSAIDs) [27] and traditional herbal medicines [28], were ascertained through questionnaires administered during the SEARCH-CKD sub-study home visits. Key additional factors that were captured included household wealth index/score (categorized as quintiles) [29], education (no formal education, primary school, or secondary school and beyond), and health habits including smoking status (never, current, or former) and current alcohol usage (any vs. none). Due to difficulty of obtaining height and weight during the study, 25.4% of our participants were missing body mass index (BMI) data. Therefore, we did not include this variable in our main analysis but conducted a sensitivity analysis among a subset of participants with available BMI data.

### Statistical analysis

We summarized the distribution of our exposures, outcomes and covariates overall, and by study region (eastern Uganda, southwestern Uganda, and western Kenya). Age was categorized into 18–29, 30–44, 45–59 and ≥ 60 years at the `time of eGFR and proteinuria measurement. To estimate the population-level prevalence of CKD and understand its associated risk factors, we used inverse probability of weighting to account for the sampling of households within each community and to adjust for incomplete ascertainment of CKD outcomes (i.e. serum creatinine and dipstick urinalysis) among selected participants [30, 31]. The household selection probability was estimated using empirical proportions specific to each community, and the probability of outcome measurement among selected participants was estimated with logistic regression controlling for age, gender, HIV serostatus, relationship to head of household, and region.

With these weights, we used multivariable log-link Poisson regression models with robust standard errors to estimate the adjusted CKD prevalence ratios [32]. Sociodemographics; health habits; history of diabetes, hypertension, or HIV; and use of NSAIDs or traditional herbal medicines have all been previously reported to be associated with CKD [8, 27, 28, 33], and thus were included *a priori* in in the regression models. A *P*-value <0.05 indicated statistical significance. Stata version 15 (StataCorp, College Station, Texas, USA) was used for all analyses.

### Ethics

Institutional review boards of Makerere University, Uganda National Council of Science and Technology (Kampala, Uganda), Kenya Medical Research Institute (Nairobi, Kenya), and the University of California, San Francisco (San Francisco, CA, USA) approved the study protocol. All participants provided signed informed consent in their preferred language.

## Results

### Population characteristics

The SEARCH-CKD sub-study completed home-visits on 3,686 residents from 22 rural communities in Uganda and Kenya. Ten communities from the parent SEARCH study were not included due to resource limitations, and 223 participants (6%) were missing serum creatine or urinalysis results (S1 Table). Table 1 shows underlying population characteristics of adults residing in rural Uganda and Kenya based on weighted SEARCH-CKD participants. The estimated mean age of these adults living in rural East Africa was 38 (standard error [SE] 0.4) years, and 52.4% (95% confidence interval [CI] 49.7–55.1%) were women. The population-weighted prevalence of HIV, diabetes and hypertension were, 9.4% (95% CI 8.5–10.5%), 3.8% (95% CI 2.9–4.8%) and 16.9% (95% CI 15.2–18.8), respectively. NSAID and traditional medicine use was estimated to be 48.4% (95% CI 45.7–51.0%) and 27.9% (95% CI 25.6–30.3%), respectively (with regional variations as shown in Table 1).

**Table 1:** Population characteristics of adults in rural East Africa based on weighted SEARCH-CKD participants.

	Eastern Uganda N = 1169	Southwestern Uganda N = 974	Western Kenya N = 1543	Total N = 3686
	% (95% CI)	% (95% CI)	% (95% CI)	% (95% CI)
Female	51.2 (46.4–56.0)	51.7 (45.9–57.4)	54.2 (50.5–57.8)	52.4 (49.7–55.1)
Age categories				
18–29	46.3 (41.4–51.3)	34.3 (28.6–40.5)	31.0 (27.4–34.9)	37.3 (34.5–40.2)
30–44	26.6 (23.0–30.5)	37.1 (31.8–42.6)	37.9 (34.6–41.4)	33.7 (31.3–36.2)
45–59	17.4 (14.6–20.5)	18.3 (15.1–22.0)	17.4 (15.3–19.8)	17.7 (16.1–19.4)
≥ 60	9.7 (7.8–12.1)	10.3 (8.0–13.2)	13.6 (11.7–15.7)	11.3 (10.1–12.6)
Education level				
No formal education	16.7 (13.8–19.9)	14.3 (11.6–17.5)	4.7 (3.7–6.0)	11.6 (10.3–13.1)
Primary school	59.6 (54.8–64.3)	59.0 (53.3–64.4)	82.1 (79.1–84.7)	67.6 (65.0–70.1)
Secondary school and beyond	23.7 (19.5–28.6)	26.8 (21.8–32.4)	13.2 (10.8–16.1)	20.8 (18.5–23.3)
Wealth Index/score^⟘^				
1^st^ quintile	14.1 (11.5–17.0)	21.6 (17.6–26.3)	12.2 (9.9–14.8)	15.6 (13.8–17.4)
2^nd^ quintile	19.5 (16.1–23.3)	22.0 (17.7–27.0)	12.2 (10.1–14.8)	17.6 (15.6–19.7)
3^rd^ quintile	21.1 (17.3–25.6)	19.3 (15.2–24.2)	22.1 (19.3–25.2)	21.0 (18.8–23.3)
4^th^ quintile	25.0 (20.8–29.6)	15.2 (11.7–19.7)	24.0 (21.1–27.1)	21.8 (19.7–24.1)
5^th^ quintile	20.4 (16.6–24.8)	21.9 (17.1–27.6)	29.5 (26.4–32.9)	24.1 (21.8–26.6)
Farmer	67.4 (62.2–72.3)	66.1 (60.1–71.6)	47.8 (44.4–51.2)	59.9 (57.2–62.5)
Tobacco use				
Current	2.5 (1.6–3.9)	10.3 (7.3–14.4)	4.7 (3.6–6.3)	5.6 (4.5–6.9)
Past	2.7 (1.6–4.6)	11.2 (8.4–14.7)	3.9 (2.7–5.4)	5.6 (4.5–6.8)
Any current alcohol use	13.4 (10.3–17.2)	18.9 (14.2–24.6)	3.5 (2.4–5.1)	11.3 (9.4–13.5)
Body Mass Index				
Underweight (< 18.5 kg/m^2^)	19.7 (15.6–24.7)	15.8 (11.7–21.0)	13.7 (11.1–16.9)	16.5 (14.3–19.0)
Normal (18.5–24.9 kg/m^2^)	62.8 (57.6–67.8)	66.0 (60.2–71.3)	71.2 (67.4–74.8)	66.6 (63.8–69.4)
Overweight (25.0–29.9 kg/m^2^)	14.0 (11.1–17.5)	14.6 (11.3–18.7)	11.7 (9.5–14.4)	13.4 (11.7–15.3)
Obese (≥30.0 kg/m^2^)	3.5 (2.2–5.5)	3.6 (2.2–6.1)	3.3 (2.2–4.9)	3.5 (2.7–4.5)
HIV-positive	3.1 (2.1–4.5)	6.9 (5.2–9.1)	17.6 (15.8–19.6)	9.4 (8.5–10.5)
Diabetes mellitus	4.2 (2.7–6.6)	5.8 (4.1–8.1)	1.7 (1.1–2.7)	3.8 (2.9–4.8)
Hypertension	18.8 (15.7–22.3)	20.6 (16.8–25.1)	12.3 (10.4–14.6)	16.9 (15.2–18.8)
Any NSAID use over the previous 90 days	33.9 (29.5–38.6)	49.2 (43.5–55.0)	61.7 (58.1–65.1)	48.4 (45.7–51.0)
Any traditional medicine use over the previous 90 days	22.3 (18.9–26.2)	43.7 (38.2–49.4)	20.7 (17.9–23.8)	27.9 (25.6–30.3)

^⟘^Wealth index/score (divided in quintiles) was calculated using principal components analysis based on ownership of livestock and other household items items [29].

NSAID: nonsteroidal anti-inflammatory drugs

### Proteinuria and eGFR

The estimated population-based prevalence of proteinuria was 5.4% (95% CI 4.4–6.6%). Proteinuria prevalence was estimated to be highest in eastern Uganda at 11.2% (95% CI 8.9–13.9%), compared to 3.5% (95% CI 1.9–6.4%) in southwestern Uganda and 1.4% (95% CI 0.8–2.3%) in western Kenya, respectively (Table 2).

**Table 2:** Prevalence of Kidney function and proteinuria categories, by region and overall.

	Eastern Uganda	Southwestern Uganda	Western Kenya	All
	Prevalence, % (95% CI)	Prevalence, % (95% CI)	Prevalence, % (95% CI)	Prevalence, % (95% CI)
eGFR ≥ 90	79.9 (76.3–83.0)	87.7 (84.3–90.4)	79.8 (77.3–82.1)	82.1 (80.3–83.8)
eGFR 60–89	18.6 (15.5–22.1)	11.8 (9.1–15.1)	17.5 (15.4–19.9)	16.2 (14.7–18.0)
eGFR < 60	1.6 (0.8–3.0)	0.5 (0.2–1.5)	2.6 (1.8–3.8)	1.7 (1.2–2.3)
Proteinuria	11.2 (8.9–13.9)	3.5 (1.9–6.5)	1.4 (0.8–2.3)	5.4 (4.4–6.6)
CKD				
eGFR < 60 or proteinuria	12.5 (10.1–15.4)	3.9 (2.2–6.8)	3.7 (2.7–5.1)	6.8 (5.7–8.1)

CKD: Chronic kidney disease

eGFR: estimated glomerular filtration rate in ml/min/1.73m^2^

We estimated that the vast majority of individuals in East Africa (98.3%) have an eGFR ≥60 ml/min/1.73 m^2^, with a weighted mean eGFR of 108 ml/min/1.73 m^2^ (SE 0.5).

### Prevalence of CKD

The estimated population-weighted prevalence of CKD was 6.8% (95% CI 5.7–8.1%). However, the prevalence varied greatly by region, with eastern Uganda having the highest prevalence at 12.5% (95% CI 10.1–15.4%) compared with 3.9% (95% CI 2.2–6.8%) in southwestern Uganda and 3.7% (95% CI: 2.7–5.1%) in western Kenya (Table 2). We estimated that of the individuals with CKD in this region, the majority (75.8%) have stage 1 or 2, while 24.2% have stage 3–5 CKD.

### Risk factors for prevalent CKD

In our univariable analyses, the factors most strongly associated with prevalent CKD were being older, residence in eastern Uganda, lack of formal education, past smoking status and hypertension (Table 3).

**Table 3:** Univariable and multivariable association of risk factors with prevalent CKD.

	Unadjusted Prevalence Ratio (95% CI)	Adjusted Prevalence Ratio♦ (95% CI)
Region		
Eastern Uganda	3.33 (2.29–4.86)	3.88 (2.56–5.89)
South West Uganda	1.03 (0.53–1.98)	1.16 (0.48–2.79)
Western Kenya	Reference	Reference
Female	1.25 (0.86–1.81)	0.98 (0.64–1.51)
Age (Years)		
18–29	Reference	Reference
30–44	1.03 (0.58–1.83)	1.15 (0.63–2.11)
45–59	2.05 (1.15–3.63)	1.92 (1.05–3.54)
≥ 60	3.66 (2.12–6.32)	3.52 (1.89–6.54)
Education level		
No formal education	2.38 (1.28–4.45)	1.24 (0.52–2.95)
Primary school	1.12 (0.62–2.02)	1.11s (0.51–2.42)
Secondary school and beyond	Reference	Reference
Wealth Index		
1^st^ quintile (Least Wealth)	Reference	Reference
2^nd^ quintile (Less Wealth)	1.02 (0.62–1.68)	1.08 (0.67–1.73)
3^rd^ quintile (Middle Wealth)	0.71 (0.42–1.20)	0.69 (0.41–1.16)
4^th^ quintile (More Wealth)	0.91 (0.52–1.59)	0.90 (0.52–1.56)
5^th^ quintile (Most Wealth)	0.94 (0.55–1.62)	1.10 (0.59–2.06)
Farmer	1.41 (0.94–2.13)	0.87 (0.53–1.44)
Smoking status		
Current smoker	1.20 (0.59–2.44)	1.32 (0.67–2.60)
Past smoker	1.79 (1.07–2.98)	1.68 (0.93–3.05)
Any alcohol use	0.82 (0.47–1.43)	0.64 (0.34–1.18)
HIV Positive	1.05 (0.79–1.39)	1.58 (1.11–2.24)
Diabetes	1.78 (0.85–3.72)	0.86 (0.35–2.11)
Hypertension	1.68 (1.17–2.41)	1.05 (0.74–1.50)
Any NSAIDs use	0.70 (0.49–1.00)	0.83 (0.57–1.22)
Any traditional medicine use	0.92 (0.61–1.39)	0.96 (0.62–1.51)

^♦^Multivariable model included region, demographics, wealth index, farming occupation, history of diabetes, hypertension, HIV, use of NSAIDs and traditional herbal medicines

CKD: Chronic kidney disease. NSAID: nonsteroidal anti-inflammatory drugs

In our fully adjusted multivariable model, age ≥60 years (adjusted prevalent ratio [aPR] = 3.5 [95% CI 1.9–6.5] compared with age 18–29), and residence in eastern Uganda (aPR = 3.9 [95% CI 2.6–5.9]) remained strongly associated with prevalent CKD. While HIV seropositivity was also associated with prevalent CKD in the multivariable model (aPR = 1.6 [95% CI 1.1–2.1]), there were no significant associations between hypertension, diabetes, NSAIDs or traditional medicine use with prevalent CKD (Table 3).

Notably, we estimated that two-thirds of individuals (65.8%) with CKD in East Africa did not have HIV, diabetes or hypertension.

### Other urine dipstick characteristics

**Fig 2:**
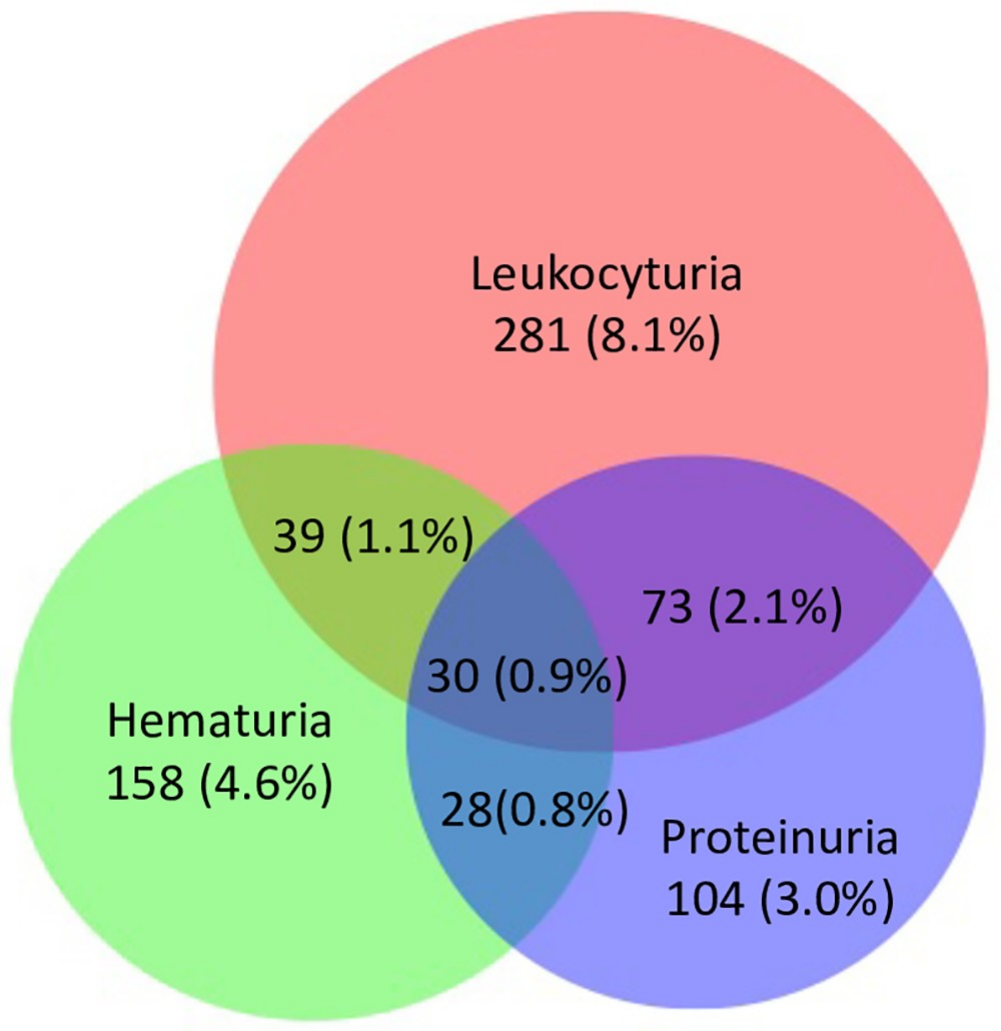
Venn diagram showing relationships between leukocyturia, proteinuria, and hematuria among SEARCH-CKD participants with both serum creatinine and urine dipstick results (N = 3463).

Among the dipstick urinalyses, (N = 3463), 7.4% had hematuria and 12.2% had leukocyturia (leukocyte esterase positive) without positive nitrite. Fig 2 shows the overlap between proteinuria, hematuria and leukocyturia. Most participants with leukocyturia did not have hematuria or proteinuria. There also was minimal overlap between hematuria and proteinuria.

### Sensitivity analysis

In our sensitivity analysis using the CKD-EPI equation with the black race coefficient to estimate GFR, 99% of individuals had an eGFR ≥60 ml/min/1.73 m^2^, with a mean eGFR of 126 ml/min/1.73 m^2^ (SE 0.6). Because most of the CKD cases we identified were based on proteinuria, the overall population-weighted prevalence of CKD was very similar at 6.0% (95% CI 5.0–7.3%). In terms of risk factors, observed patterns and strengths of associations were similar.

In our sensitivity analysis among a subset of participants with available BMI we observed that being overweight was associated with CKD (aPR = 1.8 [95% CI 1.1–3.0] compared with normal BMI). There was no association of being underweight nor obese with CKD (aPR = 0.9 [95% CI 0.6–1.4], and aPR = 1.8 [95% CI 0.7–4.3] respectively, when compared with normal BMI).

## Discussion

The SEARCH-CKD study included 3,686 participants from 22 rural communities, spanning three regions in two East African countries. To our knowledge, this study represents one of the largest population-based study of CKD in sub-Saharan Africa. Given an estimated mean age of 38 years among these adults in East Africa, our finding of a community-based CKD prevalence of 6.8% in this young population is concerning. Moreover, the prevalence of CKD varied greatly by region, with more than a 3-fold higher prevalence in eastern Uganda. CKD prevalence was largely driven by a high prevalence of proteinuria, and most participants had preserved kidney function as estimated by the CKD-EPI equation.

Proteinuria has been shown repeatedly to be associated with increased risk of progression to ESRD [33–36] and other adverse outcomes, including cardiovascular disease and premature death [37–39]. Early detection of proteinuria, and management of its modifiable risk factors may slow progression to more advanced stages of CKD, including ESRD. This is an attractive strategy to decrease morbidity and mortality associated with advanced CKD in sub-Saharan Africa, where access to renal replacement therapy is scarce [40–42].

Although comparing available studies is difficult due to differences in CKD definition, our results are consistent with two other community-based studies from East Africa, one from central Uganda [43], and another from northern Tanzania [44]. These two studies yielded a similar prevalence of CKD (7–10%), which was also predominantly driven by proteinuria [43, 44]. In West and Central Africa, community-based CKD prevalence estimates have ranged from 6–19% [45–49], with highest prevalence reported in Southwestern Nigeria [49]. However, these studies were limited to one geographical region within each country. In contrast, the SEARCH-CKD study extends our knowledge by examining multiple communities in more than one country using a pre-defined research protocol, and shows that even within a country, there may be significant differences in CKD prevalence. Future efforts to better understand geographic variations in CKD prevalence may shed important light into disease pathophysiology and identify promising strategies to mitigate the health burden of CKD.

It is interesting to speculate whether differences in the prevalence of CKD genetic risk factors across regions in sub-Saharan Africa may account for the differences in CKD prevalence. For instance, studies conducted in sub-Saharan Africa have shown that the frequency of the *APOL1* risk variants, which significantly increase the risk of CKD, varies across the continent [50]. Due to unique evolutionary pressures within sub-Saharan Africa, individuals living in sub-Saharan Africa exhibit remarkable genetic diversity [51]. For example, while the *APOL1* G1 and G2 risk variants confer protection specifically against *Trypanosoma brucei (T*.*b*.*) rhodesiense* [4], Uganda is one of the few countries where both *T*.*b*. *rhodesiense* and *T*.*b*. *gambiense* are endemic [52]. Therefore, individuals in different parts of East Africa may have variable genetic susceptibility to CKD based on regional differences in environmental pressures, but further studies are warranted.

While HIV remained an important CKD risk factor in this study, diabetes, hypertension, use of NSAIDs and traditional herbal medicines were not associated with prevalent CKD in our study. In fact, we estimated that two-thirds of individuals with CKD in East Africa do not have traditional CKD risk factors such as HIV infection, hypertension or diabetes. Our population is relatively young and would be expected to have lower prevalence of hypertension and diabetes compared to an older population, and these risk factors may become more relevant to CKD as the population ages. Currently, much remains to be unearthed about unique risk factors of CKD in East Africa. Candidate risk factors which merit study include environmental exposures such as heavy metals [53, 54], endemic infections causing glomerulonephritis, and acquired or congenital anatomic abnormalities. These risk factors may interact with genetic risk factors enriched in sub-Saharan Africa—such as the *APOL1* renal risk variants and sickle cell trait [55, 56], or other yet to be identified genetic susceptibility loci. Furthermore, these risk factors distribution may differ by geography, which can explain the geographic variation in CKD prevalence observed in this study.

A provocative (and somewhat surprising) finding we noted in the course of ascertaining proteinuria in SEARCH-CKD was how commonly hematuria and leukocyturia (without concurrent positive nitrite) occurred in this young, community-representative population—being seen in 7% and 12% of urine dipstick analyses, respectively. To our knowledge, the high prevalence of such dipstick urinalysis abnormalities among individuals in sub-Saharan African have not been previously reported. The etiologic reasons are not clear. Given the high prevalence and in the absence of positive nitrite by dipstick, simple bacterial urinary tract infection is unlikely to be the explanation. These abnormalities may reflect underlying parenchymal renal disease such as glomerulonephritis or interstitial nephritis, but we did not count them as CKD cases as we could not rule out alternative explanations such as genitourinary tuberculosis. Of note, some patients with CKD of unknown cause in agricultural communities (such as Mesoamerican nephropathy, Uddanam nephropathy or Sri Lankan nephropathy) present with leukocyturia and hematuria without proteinuria [57]. These patients have been reported to have acute or chronic interstitial nephritis on renal biopsy [58–60]. However, to our knowledge, there have been few studies of this among people living in rural Uganda and Kenya.

The major strengths of our study are, large sample size that included 3 distinct regions in two countries, population-based, and comprehensively documents the prevalence of CKD and risk factors in rural East Africa. We probed for CKD using serum creatinine as well as urine protein (and also assessed for other urinary abnormalities such as hematuria and leukocyturia which could also reflect intrinsic renal disease). We also acknowledge several limitations. First, given the study’s cross-sectional design, we could not confirm the persistence of abnormal renal parameters with repeat measurements. Second, dipstick urinalysis interpretations were not centralized or automated. However, all research assistants received extensive training and supervision during data collection, and quality assurance/control procedures were conducted daily before all measurements. Therefore, we believe that our dipstick urinalysis findings are robust. Identical dipstick kits were used in eastern Uganda and southwestern Uganda so it is unlikely that systematic errors in laboratory assessments could explain the observed differences in prevalence of proteinuria between those two regions. Third, diabetes prevalence may have been underestimated given the lack of fasting blood glucose or hemoglobin A1c measurements. Fourth, serum creatinine measurements were also not centralized; however, each of the laboratories conducted daily internal calibration and paid particular attention to this aspect of quality control. Finally, we did not probe for symptoms of urinary tract infection (e.g. dysuria) or back pain which potentially could shed light on the reasons for dipstick urinalysis abnormalities [57].

In conclusion, we observed a high prevalence of CKD, with a considerable geographic variation in a young population of rural East Africans. The high CKD prevalence was largely driven by proteinuria and provides opportunities for early interventions to delay progression of kidney disease to ESRD in an area with limited renal replacement therapy. While CKD is associated with HIV infection, two-thirds of CKD cases appear without an obvious CKD risk factor, and leukocyturia and hematuria were common. Our data highlight the urgent need for further research to better understand cause and distribution of CKD in this region.

## Supporting information

S1 Table SEARCH-CKD sampled participants. (DOCX)

S1 Fig Diagram showing number of participants with measured outcomes and missing in the SEARCH-CKD study. (DOCX)
