# Supplementary material for: The epidemiology of chronic kidney disease (CKD) in rural East Africa: A population-based study
Source: PLoS One. 2020 Mar 4;15(3):e0229649. doi: 10.1371/journal.pone.0229649 (PMC7055898; doi:10.1371/journal.pone.0229649)
Supplement: S1 Table — (DOCX) [file pone.0229649.s001.docx]

| S1 Table: SEARCH-CKD sampled participants | | | | |
| --- | --- | --- | --- | --- |
|  | Eastern Uganda  (n=1169) | Southwestern Uganda  (n=974) | Western Kenya  (n=1543) | Total  (n=3686) |
|  | n (%) | n (%) | n (%) | n (%) |
| Female ^a^ | 715 (61.2) | 641 (65.8) | 1034 (67.0) | 2390 (64.8) |
| Age categories |  |  |  |  |
| 18–29 | 260 (22.2) | 171 (17.6) | 281 (18.2) | 712 (19.3) |
| 30–44 | 424 (36.3) | 390 (40.0) | 636 (41.2) | 1450 (39.3) |
| 45–59 | 317 (27.1) | 263 (27.0) | 361 (23.4) | 941 (25.5) |
| ≥ 60 | 168 (14.4) | 150 (15.4) | 265 (17.2) | 583 (15.8) |
| Education level ^b^ |  |  |  |  |
| No formal education | 263 (22.5) | 226 (23.2) | 105 (6.8) | 594 (16.1) |
| Primary school | 671 (57.4) | 525 (53.9) | 1279 (82.9) | 2475 (67.2) |
| Secondary school and beyond | 208 (17.8) | 193 (19.8) | 146 (9.5) | 547 (14.8) |
| Wealth Index/score ^c⊥^ |  |  |  |  |
| 1^st^ quintile | 236 (20.2) | 283 (29.1) | 190 (12.3) | 709 (19.2) |
| 2^nd^ quintile | 240 (20.5) | 190 (19.5) | 185 (12.0) | 615 (16.7) |
| 3^rd^ quintile | 209 (17.9) | 186 (19.1) | 337 (21.8) | 732 (19.9) |
| 4^th^ quintile | 250 (21.4) | 150 (15.4) | 387 (25.1) | 787 (21.4) |
| 5^th^ quintile | 210 (18.0) | 137 (14.1) | 436 (28.3) | 783 (21.2) |
| Farmer ^d^ | 881 (75.4) | 802 (52.0) | 691 (70.9) | 2374 (64.4) |
| Smoking status ^e^ |  |  |  |  |
| Current smoker | 45 (3.9) | 112 (11.5) | 68 (4.4) | 225 (6.1) |
| Past smoker | 43 (3.7) | 135 (13.9) | 61(4.0) | 239 (6.5) |
| Any alcohol use ^f^ | 163 (13.9) | 142 (14.6) | 44 (2.9) | 349 (9.5) |
| Body Mass Index ^g^ |  |  |  |  |
| Underweight (< 18.5 kg/m^2^) | 158 (13.5) | 129 (13.2) | 157 (10.2) | 444 (12.1) |
| Normal (18.5 – 24.9 kg/m^2^) | 599 (51.2) | 477 (49.0) | 749 (48.5) | 1825 (49.5) |
| Overweight (25.0 – 29.9 kg/m^2^) | 144 (12.3) | 113 (11.6) | 129 (8.4) | 386 (10.5) |
| Obese (≥30.0 kg/m^2^) | 32 (2.7) | 29 (3.0) | 34 (2.2) | 95 (2.6) |
| HIV-positive ^h^ | 476 (40.7) | 391 (40.1) | 744 (48.2) | 1611 (43.7) |
| Diabetes mellitus ^i^ | 57 (4.9) | 59 (6.1) | 30 (1.9) | 146 (4.0) |
| Hypertension ^j^ | 274 (23.4) | 206 (21.2) | 224 (14.5) | 704 (19.1) |
| Any NSAID use over the previous 90 days ^k^ | 407 (34.8) | 472 (48.5) | 951 (61.6) | 1830 (49.7) |
| Any traditional medicine use over the previous 90 days ^k^ | 281 (24.0) | 400 (41.1) | 299 (19.4) | 980 (26.6) |
| Random blood sugar, mmol/L mean (SD) | 5.8 (1.6) | 6.0 (1.8) | 5.8 (1.4) | 5.9 (1.6) |
| Systolic blood pressure, mm Hg mean (SD) | 128 (20) | 129 (19) | 116 (19) | 123 (20) |
| Diastolic blood pressure, mm Hg mean (SD) | 79 (11) | 78 (9) | 72 (11) | 76 (11) |
| eGFR, ml/min/1.73m^2^ mean (SD) | 101 (22) | 106 (18) | 101 (22) | 103 (21) |
| ^⊥^Wealth index/score (divided in quintiles) was calculated using principal components analysis based on ownership of livestock and other household items items^29^  NSAID: nonsteroidal anti-inflammatory drugs  eGFR: estimated glomerular filtration rate  a-k: Missing for a. n=1 (0.03%), b. n=70 (1.9%), c. n=60 (1.6%), d. n=67 (1.8%), e. n=75 (2.0%), f. n=319 (8.7%), g. n=936 (25.4%), h. n=59 (1.6%), i. n=76 (2.1%), j. n=81 (2.2%), k. n=74 (2.0%) | | | | |
