# Supplementary material for: The epidemiology of chronic kidney disease (CKD) in rural East Africa: A population-based study
Source: PLoS One. 2020 Mar 4;15(3):e0229649. doi: 10.1371/journal.pone.0229649 (PMC7055898; doi:10.1371/journal.pone.0229649)
Supplement: S1 Fig — (DOCX) [file pone.0229649.s002.docx]

**S1Fig: Diagram showing number of participants with measured outcomes and missing in the SEARCH-CKD study**
